# Digital Technology Access and Health-Related Internet Use Among People Experiencing Homelessness in Hungary: Quantitative Survey

**DOI:** 10.2196/38729

**Published:** 2022-10-19

**Authors:** Nóra Radó, Edmond Girasek, Sándor Békási, Zsuzsa Győrffy

**Affiliations:** 1 Institute of Behavioural Sciences Faculty of Medicine Semmelweis University Budapest Hungary; 2 Health Centre Hungarian Charity Service of the Order of Malta Budapest Hungary; 3 Telemedicine Workgroup FitPuli Kft. Győr Hungary

**Keywords:** homelessness, digital technology, internet, access, internet use, homeless shelter, digital equity, mobile phone

## Abstract

**Background:**

In recent years, there has been an increase in the use of digital technology for personal health and well-being. Previous research has revealed that these technologies might provide vulnerable populations, including those who are homeless, better access to health services and thus a greater chance of more personalized care.

**Objective:**

However, little is known about the relationship between technology and health among people experiencing homelessness in Central and Eastern Europe. This study is part of a series of studies by the Digital Health Research Group at Semmelweis University (Budapest, Hungary) in cooperation with the Hungarian Charity Service of the Order of Malta; it aims to assess the existing technological resources available for the homeless population and their health-related internet use characteristics to set the ground for potential health policy interventions, enabling better access to health services by strengthening the digital components of the existing health care system.

**Methods:**

Between April 19, 2021, and August 11, 2021, a total of 662 people from 28 institutions providing social services for people experiencing homelessness in Budapest, Hungary, were surveyed about their access to digital tools and internet use patterns. For selected questions, the responses of a representative sample of the Hungarian population were used for comparison as the reference group. Chi-square tests and logistic regression analyses were performed to identify variables affecting internet use for health-related reasons.

**Results:**

The results demonstrated a considerable level of internet use in the homeless population; 52.9% (350/662) of the respondents used the internet frequently compared with 81.3% (1220/1500) of the respondents in the reference group. Among the homeless group, 69.6% (461/662) of the respondents reported mobile phone ownership, and 39.9% (264/662) of the respondents added that it had a smartphone function. Moreover, 11.2% (70/662) of the respondents had already used a health mobile app, and 34.6% (229/662) of the respondents had used the internet for medical purposes. On the basis of these characteristics, we were able to identify a broadly defined, digitally engaged group among people experiencing homelessness (129/662, 19.5%). This subpopulation was inclined to benefit from digitalization related to their personal health. Multivariate analysis demonstrated that internet use for health reasons was more significant for younger respondents, women, those with higher levels of education, and those with no chronic conditions.

**Conclusions:**

Although compared with the general population, health-related internet use statistics are lower, our results show that the idea of involving homeless populations in the digital health ecosystem is viable, especially if barriers to access are systematically reduced. The results show that digital health services have great promise as another tool in the hands of community shelters for keeping homeless populations well ingrained in the social infrastructure as well as for disease prevention purposes.

## Introduction

### Homelessness in Hungary

Homelessness is a complex set of social, economic, and health challenges at both the individual and community levels. The term itself represents a generic expression for people who live on the streets (rough sleepers), people without permanent living arrangements, or those with inadequate habitations. In Hungary, according to the law, people experiencing homelessness are persons without any registered place of residence or whose registered place of residence is the accommodation for homeless individuals [1].

Although previous research has acknowledged the difficulty in the assessment of the scale of homelessness across Europe [2], it has been noted that the number of people experiencing homelessness is increasing in the European Union [3]; approximately 700,000 people are homeless on any given day, and this number has increased by 70% in the last 10 years [1]. In Hungary, systematic resources on homeless populations are scarce, meaning that there is a lack of basic demographic studies, and no public databases are available on the estimates of the size of the group.

### Homelessness, Inequalities, and Health

The state of homelessness can be described as both a cause and a consequence of poor health status, social exclusion, and marginalization [2]. According to research, the health effects produced by homelessness include significantly higher rates of bacterial and viral infections, diabetes, hypertension, and cardiovascular disease compared with populations with adequate housing options [4]. Similar results emerged when looking at the life expectancy of homeless and general populations; on average, a decrease by 11 years for homeless men and 15 years for homeless women was measured [4].

Furthermore, earlier research suggests that despite the poor health status of homeless populations, health services designed for their treatment are often described as insufficient and limited in their accessibility, availability, and appropriateness [5]. An earlier study conducted in the United States also noted a medicalization process among homeless services and the practice of providing services for homeless individuals to conform them to specific behaviors [6]. As a result, underdiagnoses and undertreatment of health conditions are strongly prevalent [7,8], significantly underpinning the necessity to develop novel approaches and interventions to address health inequalities that have existed for decades, as such disparities lower life expectancy and strengthen social exclusion.

### Digital Tools and Digital Inclusion as Potential New Approaches

The COVID-19 pandemic has accelerated the adoption of digital technologies in health care systems in many countries that experienced various types of lockdowns between 2020 and 2022. The World Health Organization’s assessment of the European digital health landscape describes that during the COVID-19 pandemic, many digital health tools moved from being viewed as a potential opportunity to becoming an immediate necessity, and their use increased substantially [9]. The pandemic is also believed to have demonstrated that the lack of broadband access to the internet has an influence on the social determinants of health [10].

Although the expansion of the digital component of health care systems is considered a forward-looking development, it has raised accessibility issues for vulnerable strata, such as homeless populations. Physical barriers in the form of lack of access to technological equipment, as well as educational barriers in being unable to use the technology, may contribute to the inaccessibility of services and resources, further depriving a segment of the population that is already marginalized. This very possibility would negatively impact behaviors and stressors and might further contribute to poorer health outcomes for those who are digitally excluded, widening the already existent digital inequality landscape [11,12].

A systematic review analyzing studies from 2015 to 2021 with the research questions (1) “What mobile health–related technology is used by homeless populations?” and (2) “What is the health impact of mobile technology for homeless populations?” found that most homeless participants across the 17 studies included in the review owned a mobile phone or smartphone and 80% (1205/1507) owned a mobile phone. Age appeared to be a significant factor regarding ownership and use, and confirmatory responses to questions on access to mobile internet services, smartphone functions, and apps dropped significantly [11]. Heaslip et al [11] mentioned the lack of charging points, limited or no access to data traffic, and anxiety over potential theft and harassment as barriers to mobile phone use. Other barriers presented were privacy concerns and distrust in the management of data, tracking of information, the government, and the “system” [11]. Beyond physical barriers and trust issues, access to digital health might be hindered by the lack of skills required for their use. Populations at risk for limited health literacy, such as homeless populations [13], are similarly vulnerable to having challenges with digital tools [14]. Poor IT skills among homeless populations have been implicated in poor mental health outcomes [14].

However, despite existing barriers, several studies have reported the interest of the homeless population in digital health tools [11]. Atkins et al [15] noted that their study participants were positive about using a mobile phone to obtain advice and help address issues such as depression, anxiety, self-harm, abuse, substance use, emotional problems, insomnia, and stress. In all, 3 studies showed that interest in appointment and prescription reminders among homeless populations is prevalent [15-17].

### Early Examples: Attitudes Toward Digital Health Among Homeless Individuals in Hungary

As the above literature review supports, physical barriers to accessing technologies and educational barriers in relation to digital technologies might strengthen the already existing digital inequalities to the detriment of homeless population, whereas the use of the internet was shown to be significantly associated with better self-rated health in older adults [18,19] and more favorable health behaviors concerning cancer prevention [20]. Studies conducted mainly in the United States, Canada, and the United Kingdom, focusing less on continental Europe or lower-income countries, suggest these findings [11].

The main aim of this study was to examine whether these assumptions are valid in the context of Hungarian homeless population and to suggest recommendations for public health policy makers. Thus, the main research questions were whether (1) homeless populations use digital tools for health-related reasons in Hungary and (2) clearly identifiable variables, such as the institutional and social services environment, age, education, or other demographic data can be associated with such use. In the case of social institutional characteristics, we assume that existing barriers and potentials of unique institutions to digital inclusion might be considered and offered as background information for potential interventions for digital inclusion, which we aim to examine as part of the second research question.

This study fits into a broader set of research undertaken by the joint action of the Digital Health Research Group at Semmelweis University and the Hungarian Charity Service of the Order of Malta (HCSOM), aiming to analyze the relationship between digital health and homeless populations in Hungary. Previous research has studied the attitudes of homeless individuals toward telecare services, with the main finding being that trust in the general health care system leads to trust in digital health solutions [12]. This study also served as an assessment tool for analyzing the viability of a telecare system planned to be launched by the HCSOM.

## Methods

### Participating Institutions

Homelessness can be categorized using different methods; Edgar et al [21] identified 6 different groups. As for the classification and definition of “homelessness” in this study, we decided to include all individuals who had engaged with institutions providing homeless services according to the categories of the European Typology of Homelessness and Housing Exclusion, the standard used by European Union member states for reporting on homelessness and precarious housing circumstances [22].

Altogether, 6 types of institutions providing social services for homeless populations participated in the study (Table 1). Although family shelters are not considered a part of the homeless social services according to the law in Hungary (these institutions are operated under the Child Protection Act), they were included in the study based on the housing instability of their clients and the temporary nature of the provided accommodation.

**Table 1:** List and characteristics of participating institutions and social services (N=662).

Type of service	ETHOS^a^ classification	Client	Participating institutions (N=28), n (%)	Participants, n (%)
Street outreach service	1.1	Rough sleepers	4 (14)	106 (16)
Day shelter	N/A^b^	Homeless persons (no accommodation offered)	5 (17.9)	167 (25.2)
Night shelter	2.1	Homeless persons (accommodation offered only for short periods)	7 (25)	145 (21.9)
Temporary shelter	3.2-7.2	Homeless persons (accommodation offered for longer periods with a maximum of 1+1 years)	7 (25)	178 (26.8)
Temporary shelter with a focus on health improvement	3.2-7.2	Homeless persons with severe health status (accommodation offered for longer periods with a maximum of 1+1 years)	2 (7.1)	40 (6)
Family shelter	7.2	Homeless families (accommodation offered for longer periods with a maximum of 1+1 or 2 years)	3 (10.7)	48 (72.5)

^a^ETHOS: European Typology of Homelessness and Housing Exclusion.

^b^N/A: not applicable.

### The Surveying Process

The research team formulated a questionnaire (Multimedia Appendix 1) based on the Digital Inclusion Survey used in a report by Pathway, the United Kingdom’s leading homeless health care charity [23]. The original questionnaire was translated to Hungarian by 2 independent medical translators, and their versions were merged by a consensus meeting. This Hungarian draft questionnaire was adapted to the local specialties during a workshop with social workers of the HCSOM. Before administering the questionnaire to a wider population, a test survey with 10 participants was completed to check its clarity and intelligibility. The selection of test group members was managed by one of the participating social establishments. To maximize the impact of the test survey, it was requested to use a diverse group of homeless clients with respect to gender, age, health status, and type of accommodation. Subtle changes in wording were applied during the finalization of the survey material based on this feedback.

Between April 19, 2021, and August 11, 2021, the research group surveyed 662 people in Budapest, Hungary, with the cooperation of 28 institutions that provide various social services for homeless individuals. The respondents participated in the study on a voluntary basis. Our research team contacted the institutions, and their social workers asked homeless clients to fill out the questionnaires in a paper and pencil form. Social workers were allowed to help in the interpretation of questions but were not allowed to influence the answers. When a respondent was using multiple social services (eg, day and night shelter), we asked individuals to complete the questionnaire at the institution that provided the most relevant service for them to reduce duplicate responses.

The questionnaire enquired about sociodemographic data (age, gender, level of education, self-defined homelessness, and length of being homeless) and health status (frequency of medical visits, existing medical diagnoses, and self-assessment of health status). Questions 6-10 were used to gather information about health knowledge and general literacy skills, whereas questions 11-13 and 14-17 asked about access to mobile phones and the internet. Next, questions 18-21 inquired about internet use habits and questions 22 and 23 about potential barriers and enablers of internet access. Question 24 presented a set of statements about digital health literacy, and question 25 asked about mobile apps.

### Reference Group

For the questions “How frequently do you visit a medical doctor/do you use medical services?” “Do you have any chronic disease or a long-term health problem?” “Have you ever used the Internet for any purpose? If yes, have you used it in the last six months?” and “Have you ever used any health-related mobile applications?” the responses of a representative sample of the Hungarian population were used as a reference group to provide more context. This representative survey was conducted by the Digital Health Working Group of Behavioral Institute of Semmelweis University between October 5, 2021, and October 13, 2021, and consisted of responses from 1500 Hungarian people in the framework of the “E-Patients in Hungary” study [24].

### Statistical Analysis

As part of the quantitative analysis, we descriptively examined frequencies, averages, and percentage distributions. Use of technology and its various correlates (demographic variables and variables related to access to health services) were compared with a single variable analysis using Pearson chi-square test, with a significance level of *P*<.05.

In the multivariate analysis, a binary logistic regression model was used. The method was used to examine the background factors for the question “Have you ever used the internet for health reasons?” which is the dependent variable. The control variables were gender, type of institution and social service, level of education, age, frequency of medical visits, and prevalence of chronic illness. Independent variables affecting the dependent variables were selected using enter regression. The significance of the regression coefficients of the given variables was described using *P* value of the Wald. Variables with *P*<.05 were retained in the final model.

Data were analyzed using SPSS (version 26; IBM Corp) statistics software [25].

### Ethics Approval

The data collection was anonymized. Written informed consent statements were obtained in all cases, and ethics approval for the study was issued under TUKEB:133/2020 and IV/10927/2020/EKU by the Scientific Research Ethics Committee of the Medical Research Council of Hungary.

## Results

### Demographics

**Figure 1:**
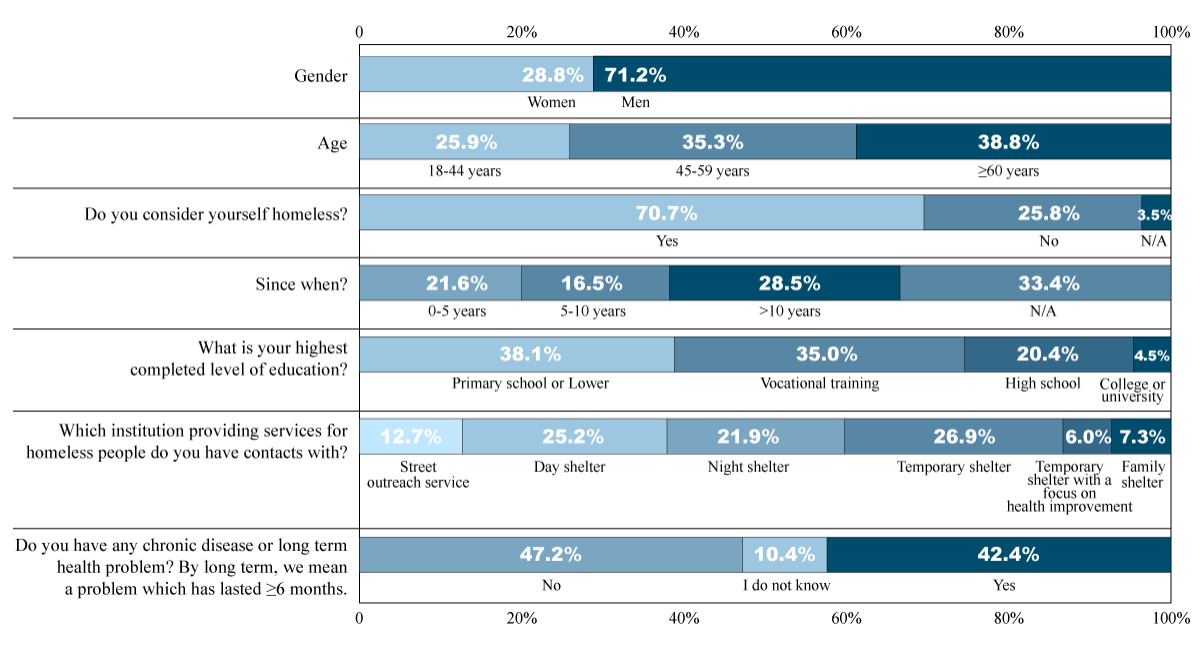
Key demographics of the homeless group. N/A: not applicable.

The research group surveyed 662 adults in Budapest, Hungary, recruited from 28 social institutions providing services for people experiencing homelessness. Of the respondents, 71.2% (459/662) were men. Of the recruited participants, 38.8% (247/662) represented the age group of >60 years, whereas participants aged 18 to 44 years accounted for only 25.9% (165/662). The mean age was 53.9 years with an SD of 13.08 years. The majority, 70.7% (468/662), considered themselves homeless, whereas 25.8% (171/662) of the respondents did not consider themselves homeless. A total of 66.6% (441/662) of respondents also indicated how long they were experiencing homelessness: 21.6% (143/662) had been homeless for 1 to 5 years, 16.5% (109/662) for 5 to 10 years, and 28.5% (189/662) for >10 years, with a mean of 11.35 years and an SD of 9.27 years. Most of the respondents had only primary education (252/662, 38.1%) or vocational training (232/662, 35%), whereas 20.4% (135/662) of the respondents had graduated high school, and 4.5% (30/662) of the respondents said they had completed their college or university education. The key demographic parameters are shown in Figure 1.

### Health Status

As key independent variables, we surveyed the health status of the respondents and compared them with the data of the reference group. A total of 16.5% (109/662) of the respondents said that they visited their physician or used health care services more than once a month, which was relatively frequent compared with the reference group, wherein 6.4% (96/1500) respondents said they visited their physician weekly, more than once a week, or more than once a month. Within the homeless group, 21.8% (144/662) of the respondents said they visited their physician every 1 or 2 months, which is almost the same as the result for the reference group (284/1500, 18.9%). The main difference was that most of the homeless group, 42.3% (280/662), visited their physician only yearly or less frequently, whereas 35.9% (539/1500) of the reference group said they used health care services 1 to 2 occasions per year, and only 13% (195/1500) of the respondents reported going to the physician’s office yearly.

Of the homeless participants, 46.1% (305/662) reported no chronic diseases or long-term illnesses requiring treatment lasting for ≥6 months, but there was only a slight difference in the distribution of those who did (274/662, 41.4%). Those who had a chronic disease listed chronic obstructive pulmonary disease, asthma, diabetes, hypertension, mental illnesses, and chronic heart conditions among others. For the reference group, 48.8% (732/1500) of the respondents responded that they had a long-term illness, whereas 51% (765/1500) said that they did not have any.

**Figure 2:**
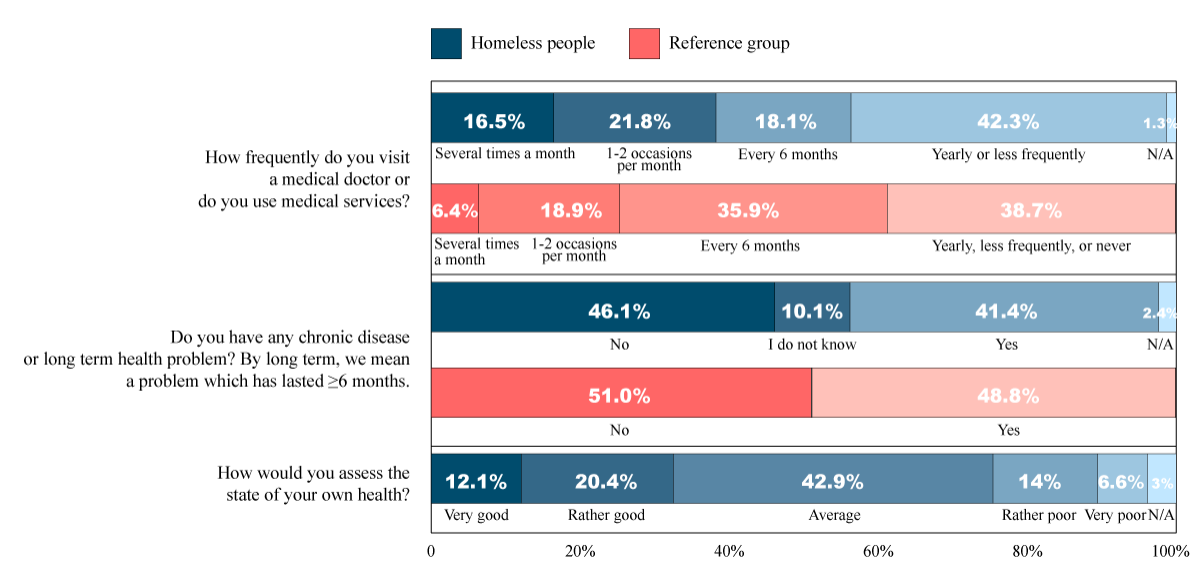
Key demographics concerning health status of the homeless group. N/A: not applicable.

Regarding the homeless group evaluating their own health, 12.1% (80/662) and 20.4% (135/662) of the respondents said “very good” or “rather good,” respectively, whereas most people (284/662, 42.9%) considered it “average.” In addition, 14% (93/662) and 6.6% (44/662) of the respondents said they considered their health “rather poor” and “very poor,” respectively (Figure 2).

When asked about what channels they were using when informing about medical issues, 20.5% (136/662) of the respondents said they were searching for it on the web. This came in third after asking the primary care physician for information (352/662, 53.1%) and the social worker in the social institution (260/662, 39.2%), which meant they might have been consulting the internet for medical purposes more often than they asked their family members or friends (108/662, 16.3%).

### Access to Technology and Web-Based Services

For the multiple-choice question, “How do you access the internet at the moment?” 98 people (98/551, 17.8%) said that they had their own smartphone with a data contract, 100 people (100/551, 18.1%) said that they had their own smartphone using a pay-as-you-go facility, 118 people (118/551, 21.4%) said that they had their own smartphone and accessed the internet via free Wi-Fi hotspots, 136 people (136/551, 24.7%) said that they accessed the internet through a publicly available PC in social institutions or shelters, only 15 people (15/551, 2.7%) said that they had their own PC, and 84 people (84/551, 15.2%) responded with “Other.” In the latter category, answers included the use of other people’s phones, “internet cafés,” or ownership of a tablet, but a frequent response was that they had no means to access it, they did not care, or they did not use it. Only a few people access the internet in multiple ways (70/662, 10.6% in 2 ways, 12/662, 1.8% in 3 ways, and 4/662, 0.6% in 4 ways), while more than half of the respondents have access to it in only one way (359/662, 54.2%) or in no way (217/662, 32.8%).

**Figure 3:**
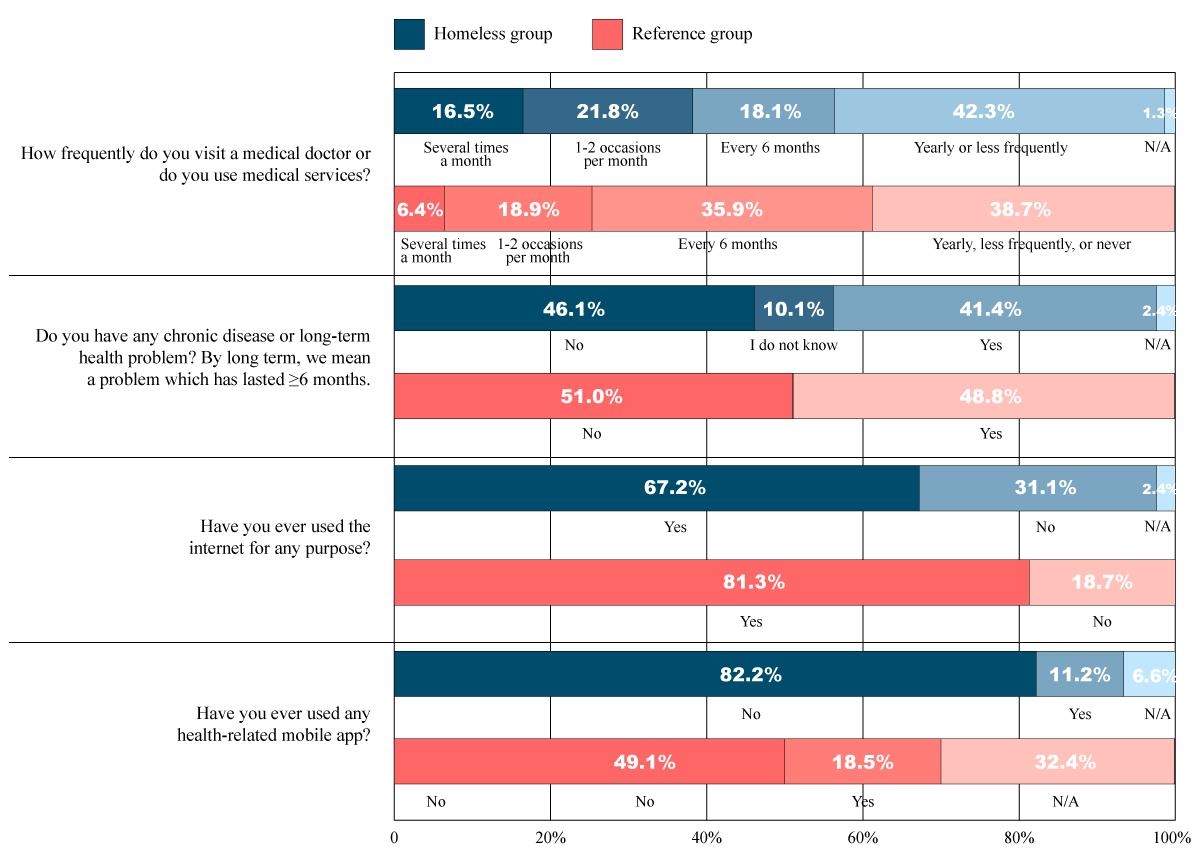
Health and internet use characteristics of the homeless and reference groups. N/A: not applicable.

In the reference group, 81.3% (1220/1500) of the respondents said that they used the internet frequently, whereas in the homeless group, 67.2% (445/662) of the responses were affirmative when asked if they ever used it for any purpose (Figure 3). Of those who used it, 52.9% (350/662) said they had used it in the past 6 months. However, daily use was significantly less, 34.6% (229/662), and an additional 10.6% (70/662) of the respondents said that they were using it more times a week. No correlation with age, type of institution and social service, gender, education, length of homelessness, or frequency of medical visits was found after cross-tabulation.

Most respondents of the homeless population (461/662, 69.6%) said that they owned a mobile phone. In addition, 39.9% (264/662) of the respondents also said that their mobile phone had a smartphone function, and 11.2% (74/662) of the respondents of the homeless group said that they had used at least one mobile health (mHealth) app, whereas this ratio was 18.5% (277/1500) in the reference group. In the homeless group, those who responded positively to the questions mentioned using apps for step counting, accessing emergency help, obtaining relevant medical information, and providing health data. mHealth apps were associated with 2 variables. Chi-square test results were significant for the type of institution and social service (*P*=.02) and frequency of medical visits (*P*=.03), meaning that mHealth apps were more frequently used in temporary shelters than in any other type of institution and social service, and with an increasing frequency of medical visits, the frequency of mHealth app use also increased.

For the question of how experienced they considered themselves when it came to internet use, 10% (66/662) of the respondents said “very much so,” 14.5% (96/662) of the respondents said “rather experienced,” and 21.5% (142/662) of the respondents said “mediocre,” whereas 10.3% (68/662) of the respondents considered themselves “rather not experienced,” and the most prevalent response, 35.3% (234/662), was “not at all” experienced. A total of 8.5% (56/662) did not respond to the question. When cross-tabulating self-reported technology literacy with age, education, gender, homelessness, type of institution and social service, and frequency of medical visits, chi-square tests were significant for age (*P<*.001), type of institution and social service (*P*=.01), and education (*P*=.01), meaning that with age, the level of self-reported technological literacy decreased, whereas with higher levels of education, self-identified technology literacy increased. Most of the respondents did not consider themselves as experienced technology users; this most significantly characterized the clients of temporary shelters with a focus on health improvement, whereas most experienced technology users made use of the social services of daily and family shelters.

### Barriers and Enablers of Internet Use

For the multiple-choice question, “What barriers, if any, restrict your internet use?” of the 682 responses, 210 (30.8%) said that nothing hindered it; 104 (15.2%) said there were not enough free Wi-Fi hotspots; only 46 (6.7%) said they had a smartphone, but they did not have a data contract or pay-as-you-go facility; and 52 (7.6%) said that they had internet access, but they did not know how to use the internet. Of the 682 responses, 146 (21.4%) said that they did not have a smartphone and 60 (8.8%) said that there were not enough publicly accessible PCs (eg, in institutions providing social services). In addition, of the 682 responses, 64 (9.4%) said that they could not access the internet anywhere.

For the question, “What would help you use the internet more?” of the 598 responses, 145 (24.2%) wished to have a smartphone, 110 (18.4%) responded better access (they had a smartphone but did not have an available internet connection option), another 56 (9.4%) also responded better access (they used PCs in institutions providing social services, but only a limited number of devices were available), 135 (22.6%) responded more knowledge (they did not know how to use the internet, and it would have helped if they could get assistance); however, for most people, 152 respondents (25.4%), the question was not relevant as they already used the internet as much as they wanted.

### Health-Related Internet Use

For the question, “Have you ever used the internet for health reasons?” 34.6% (229/662) of the homeless population said that they did. In the reference group, 10.7% (160/1500) used it every day, 18.4% (276/1500) weekly, 18.2% (273/1500) monthly, and 24% (360/1500) less, encompassing 71.3% (1069/1500) of the representative sample. This means that the general population used the internet for medical purposes more than twice as frequently as the homeless population.

When cross-tabulating with gender, age, type of institution and social service, education, frequency of medical visits, and self-evaluation of health status, chi-square tests were significant for gender (*P*=.007), age (*P*<.001), and frequency of medical visits (*P*=.01), meaning that younger women respondents and those who went to the physician’s office more frequently tended to use the internet more frequently for health-related issues.

### A Digitally Engaged Group of People Experiencing Homelessness

In the course of our analysis, we found a specific subpopulation in the sample identified as a “digitally engaged group of people experiencing homelessness.” The members of this group were specific in the sense that they did not need further digital inclusion. This group was selected for further analysis based on the following inclusion criteria.

**Figure 4:**
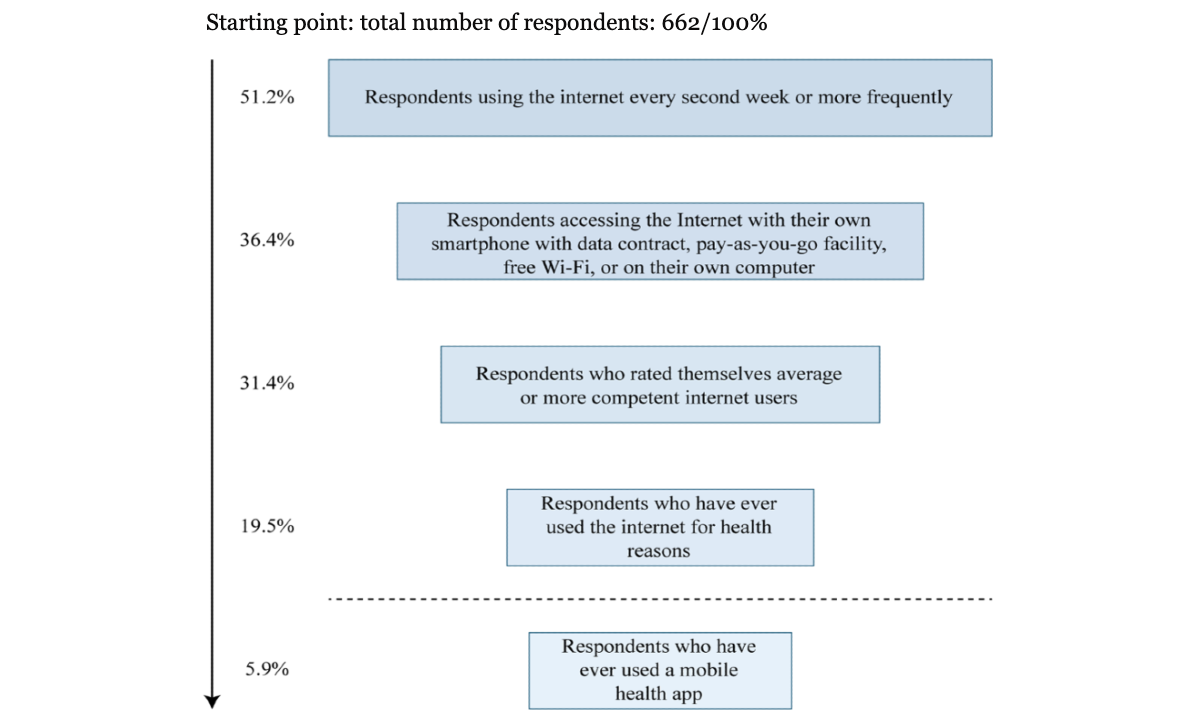
Flowchart for selecting the digitally engaged group of people experiencing homelessness.

First, we selected respondents who said that they were using the internet at least every second week (339/662, 51.2%). In the next step, we asked the respondents who reported smartphone ownership with data contract, pay-as-you-go facility, or free Wi-Fi or computer or tablet ownership to the question “How do you currently access the internet?” (241/662, 36.4%). We then filtered out the respondents who did not have a sense of being an average or more competent internet user (208/662, 31.4%). Furthermore, we selected those who responded “yes” to the question whether they had ever used the internet for health-related reasons (129/662, 19.5%). We also considered filtering the subpopulation based on the question “Have you ever used any health-related mobile application?” but as only 18.5% (277/1500) in the reference group responded positively to the question, we expected a significantly lower number in the homeless population, bordering analyzability. In contrast, the low number in the reference population indicates that mHealth app use is not necessarily meaningfully associated with overall health-related digital engagement. Thus, we created 2 subpopulations, a more broadly defined and a more strictly defined group, and analyzed their characteristics separately (Figure 4).

When the selected subgroup included 19.5% (129/662) of the total homeless population, significantly more women were included in the subpopulation (47/129, 36.4%) than the original population (186/662, 28.8%). When cross-tabulating with gender, age, education, frequency of medical visits, prevalence of chronic illnesses, and type of institution and social service, chi-square test results were significant for the prevalence of chronic illness (*P*=.047); therefore, respondents with chronic illnesses were more likely to use the internet frequently for health-related reasons. Although the institutional setting was not an associative variable, temporary shelters (40/129, 31%) and day and night shelters (28/129, 21.7% and 22/129, 17%, respectively) housed most respondents in the subpopulation (90/129, 69.7%).

Of the 662 participants, we filtered out those who had never used a health-related mobile app (Figure 4). The selected subgroup included 5.9% (39/662) of the respondents of the total studied population. The gender ratio became balanced, which means that more women (14/39, 36%) were included in the subgroup than in the original population (186/662, 28.8%). When cross-tabulating with gender, age, education, frequency of medical visits, prevalence of chronic illnesses, and type of institution and social service, the chi-square test results were significant for the institutional setting (*P*=.03) and education (*P*=.04), which means that digital engagement of a homeless person tended to depend on the type of homeless shelter the respondent frequented, and respondents with higher levels of completed education tended to be more digitally engaged.

### Multivariate Analysis

Chi-square test results showed that gender, age, and frequency of medical visits were associated with health-related internet use; however, to analyze which demographic or health status variables influenced health-related internet use, a binary logistic regression model was necessary.

The dependent variable was health-related internet use, and we entered gender (1=woman and 2=men), age (as a continuous variable), type of institution and social service (6 categories), education (4 categories), frequency of medical visits, and the prevalence of chronic conditions in the model.

The logistic regression model was found to be significant (Nagelkerke *R*^2^=0.154). After controlling for all the abovementioned variables, we found that health-related internet use showed a strong dependency on age and a statistically significant association with gender, level of education, and the prevalence of chronic conditions (*P*<.05). This means that younger homeless women who did not have any chronic conditions tended to use the internet more for health-related issues (Table 2).

**Table 2:** Results of the logistic regression model (Nagelkerke R^2^=0.154)^a^.

	B (SE)	Wald test (*df*)	*P* value	Exp (B)
Gender (1=female; 2=male)	−0.480 (0.222)	4.660 (1)	.03	0.619
What is your highest completed level of education?	—^b^	9.186 (3)	.03	—
What is your highest completed level of education? (1=primary school)	0.458 (0.483)	0.899 (1)	.34	1.581
What is your highest completed level of education? (2=vocational training)	−0.191 (0.480)	0.158 (1)	.69	0.826
What is your highest completed level of education? (3=high school)	−0.141 (0.495)	0.081 (1)	.78	0.869
How frequently do you visit a medical doctor or do you use medical services?	0.155 (0.099)	2.453 (1)	.12	1.168
Do you have any chronic disease or a long-term health problem? By long-term, we mean a problem which has lasted six months or longer.	−0.481 (0.238)	4.077 (1)	.04	0.618
Age	0.049 (0.009)	30.033 (1)	<.001	1.050
Which institution providing services for homeless people do you have contacts with?	—	3.607 (5)	—	—
Which institution providing services for homeless people do you have contacts with? (1=outreach service)	0.606 (0.458)	1.752 (1)	.19	1.833
Which institution providing services for homeless people do you have contacts with? (2=day shelter)	0.356 (0.397)	0.804 (1)	.37	1.428
Which institution providing services for homeless people do you have contacts with? (3=night shelter)	0.058 (0.431)	0.018 (1)	.89	1.059
Which institution providing services for homeless people do you have contacts with? (4=temporary shelter)	0.109 (0.434)	0.063 (1)	.80	1.115
Which institution providing services for homeless people do you have contacts with? (6=family shelter)	0.223 (0.585)	0.145 (1)	.70	1.249
Constant	−2.052 (0.838)	6.002 (1)	.01	0.128

^a^Dependent variable: Do you ever use the Internet for health reasons? (0=no; 1=yes).

^b^Not available.

## Discussion

### Principal Findings

Homeless adults experience an early onset of geriatric conditions, a complex set of chronic diseases, and premature mortality [26,27], as their access to adequate health care services is generally poor. Such disparities lower life expectancy and strengthen social exclusion. To mitigate health inequalities among homeless populations, digital technology [12], a new health determinant, can be considered on a broader scale. In a previous study by the Digital Health Research Group [12] at Semmelweis University that examined the attitudes and openness of homeless individuals regarding telecare in a Hungarian sample, a significant fraction of people experiencing homelessness with mid- or long-term residency in homeless shelters was open to the use of telecare via live web-based video consultation. As a step forward in assessing the feasibility of launching a comprehensive telehealth project and disseminating other well-being programs, the research team conducted this survey assessing existing access to digital platforms (smartphones and internet) and barriers in both physical and educational spaces among homeless populations.

On the basis of our findings, the surveyed homeless population showed an aptitude toward health-related technology use and had partial access to digital tools. Overall, the results respond to our first research question positively, that is, homeless populations use digital tools for health-related reasons.

A significant proportion of respondents had a mobile phone (461/662, 69.6%), and a lower but still significant number of respondents possessed a smartphone (264/662, 39.9%). These findings are congruent with the results presented in the literature, although according to our findings, the ownership of devices and access to the internet lag behind that of Western countries. In 2013, McInnes et al [28], in a systematic review, found that mobile phone ownership ranged from 44% to 62%, computer ownership from 24% to 40%, computer access and use from 47% to 55%, and internet use from 19% to 84% in this population. In 2017, Rhoades et al [29] found that the vast majority of homeless individuals (94%) owned a cell phone, more than half owned a smartphone, and 51% accessed the internet on their cell phones. One-third of the participants reported no internet use in the past 3 months [29]. In 2021, Thurman et al [30] analyzed feasibility studies related to mHealth interventions among people experiencing homelessness and found that 52% of the participants (n=31) reported having a personal cell phone, and of those with phones at baseline, the majority (87%) reported that their phones were capable of SMS text messaging, picture messaging, and mobile app use.

Our results showed that people experiencing homelessness turn to their family physician and social workers the most frequently for help with medical issues, but their third most frequent choice is the internet (20.5%), even before asking family members or friends. In total, 34.6% (229/662) of the respondents said they had used the internet for medical purposes, and 11.2% (74/662) of the respondents had already used a medical mobile app.

In addition, we have to consider technological limitations. The first iPhone was launched in 2007, which introduced the concept of smartphones, the spread of smartphone-based internet use, and personalized web-based searches. Technological adoption is slower in lower socioeconomic groups, and previous studies found that rates of smartphone and internet use among homeless populations were lower than those among housed, low-income adults of any age [31], which might explain the generally lower internet use statistics for this specific group. This is in line with the findings of Von Holtz et al [32] showing that, while experiencing homelessness, participants experienced a 68% less likelihood to access the internet than when they were housed; however, our main results show that the idea of involving homeless populations in the digital health ecosystem can already be based on solid use patterns, which can be further extended.

### Age as a Key Predictor of Health-Related Internet Use

On the basis of our findings, the response to our second research question, that is, clearly identifiable variables, above all institutions and social services, and beyond that, age, education, or other demographic data can be associated with health-related internet use, had to be partially rejected. Neither chi-square tests nor the binary regression model showed statistically significant results. The type of institutional access and social services provided did not relate to access and use of digital tools and the internet, except for the digitally engaged subgroup. In contrast, our logistic regression model showed that age, gender, level of education, and prevalence of chronic conditions are variables that statistically significantly influence health-related internet use.

In line with our results, Harris et al [33] found age to be a key sociodemographic variable affecting the use of technology by homeless individuals. The participants of that study felt that the shift in the United Kingdom to more digital social services had assumed that users were well versed with IT, although this may not be the case.

Although age seemed not to play a key factor in homeless individuals accessing technology, as most of the respondents had a mobile phone (461/662, 69.6%), mostly representing the age group of >60 years, it might be a crucial factor when it comes to their own perception of competence in using web-based services and health-related internet use. Younger respondents (age group 18-44 years) considered themselves rather competent, whereas older respondents (age groups 45-59 years and >60 years) did rather not or did not at all consider themselves competent when it came to using the internet. Moreover, the regression model showed that the younger a homeless respondent was, the more likely they were to use the internet for health-related reasons.

### Gender, Level of Education, and Prevalence of Chronic Conditions

The regression model showed that gender was an explanatory factor when it came to health-related internet use, which means that women in the homeless group tended to use digital tools mainly for health-related purposes. This is congruent with the trends in the general population, as Resch et al [34] found that women were more engaged in using the internet to search health-related information in Germany (n=1006), and Rising et al [35] through the 2017 and 2018 National Cancer Institute Health Information National Trends Survey (n=6789) found that in the United States, women were more likely than men to use digital health tools. As a noteworthy limitation, it has to be mentioned that women were almost 2.5 times more underrepresented in the sample (186/662, 28.8%), which might have influenced mHealth use patterns along gender lines.

Regarding the level of education, those who had completed higher levels of education were more inclined to use digital health tools, although only 4.5% (30/662) of the sample said they had completed college or university education, which, similar to the gender composition of the sample, might influence use patterns. In contrast, this finding is congruent with the self-assessment of technological literacy. Chi-square test results were significant for education (*P*=.01) when cross-tabulating with self-assessment of digital competencies, meaning that with higher levels of education, the sense of technology literacy increases, which might result in more frequent use.

Concerning the prevalence of chronic conditions, the results showed that homeless individuals without chronic diseases or any long-term illnesses tended to use the internet more for health-related purposes, which might originate from the pattern that those who were more concerned about their own health tended to use a diverse tool kit for health care and well-being, including digital tools, whereas those with serious chronic illnesses might tend to neglect their state because of their struggle to accommodate basic human needs or lack of resources for accessing care [36].

Overall, the results of the regression model were in line with trends in the general population: younger and more educated people tend to use digital health tools [37,38], and this finding means that in the course of planning health care interventions for homeless populations, patterns observed in the general population might be taken as a base for further action.

### Digitally Engaged Homeless Subpopulation

The homeless population was a diverse group in terms of health-related internet use and access to digital tools, with a significant number of digitally engaged participants. When analyzing the data, the research team found 2 broadly interpretable digitally engaged homeless subpopulations: a subpopulation without health-related mobile app use (129/662, 19.5%) and another with such use (39/662, 5.9%). Generally speaking, both digitally engaged groups included more women and younger respondents than the homeless population, which was in line with the findings of the regression model. The overall results were also congruent with previous literature stating that low-income populations rely on smartphones rather than computers for internet access; the latter was less frequent than owning a smartphone in our sample as well [31].

A chi-square test on the association between demographic factors and the more broadly defined subgroup showed that the type of institution and social service as well as the level of education—the higher the level of completed education, the more substantial digital engagement—mattered as factors for becoming digitally engaged. Temporary shelters (40/129, 31%) and day and night shelters (28/129, 21.7% and 22/129, 17%, respectively) housed most respondents in the subpopulation (90/129, 69.7%), which means that long-term living conditions seem to be associated with digital inclusion. The same pattern emerged in the more strictly defined subgroup; a chi-square test on the association between demographic factors showed that only the type of institution providing social services mattered as a factor for becoming digitally engaged. Almost half of the selected subgroup used temporary shelters, whereas very few digitally engaged users were found among rough sleepers and those who used emergency accommodations.

### Barriers and Enablers of Internet Use

Rice et al [39] reported that mobile phones can facilitate communication with family or friends and provide social support, which in turn has been shown to be associated with more favorable health outcomes [40]. In contrast, two-thirds of the participants of a cohort of 350 adults experiencing homelessness aged >50 years in Oakland, California, reported using their phones to communicate with their health care providers, suggesting both interest and feasibility [31].

However, several studies have shown homeless population’s interactions with technology to be significantly affected by lack of resources and the structural constraints [33], which was also shown by our results. As the main barriers to accessing technology, respondents mentioned affordability of digital tools or data contracts, the low number of free Wi-Fi hotspots, and PCs available at social institutions. To foster internet use, a significant number of respondents suggested overcoming these barriers rather than urging the need for educational assistance.

In line with previous studies, in the context of homeless populations in Hungary, increasing public access to high-speed internet and providing discounted smartphones for high-need, low-income individuals may also increase access to the internet [41]. Moreover, Budapest lacks an adequate number of free Wi-Fi hotspots, and thus needs more of such hotspots installed [42]. As Raven et al [31] noted, private sector technology and telecommunication companies might also be incentivized to fund initiatives that increase the use of their services among underserved populations, thereby increasing access to reliable mobile technology.

### Strengths

Studies examining health and technology-related behaviors in homeless populations tended to be conducted predominantly in the United States and Canada compared with little examination of the use of technology of homeless populations in other countries [11]. Thus, as Heaslip et al [11] also noted, further research is needed in the United Kingdom, Europe, and lower-income countries. This study aims to fill that gap by examining the accessibility and use of health-related technology in Central and Eastern Europe, more specifically in Hungary.

Compared with other studies that examine homeless populations in specific areas, the sample size of this study (N=662) is considered notable and large enough to draw statistically significant conclusions.

### Limitations

The study sample represents urban homeless populations from Budapest, Hungary, where socioeconomic conditions might differ from those living in the countryside. Homeless population recruited in our study had a connection to the social infrastructure; therefore, rough sleepers and other people who were not connected to any social initiatives were not represented.

The research team relied exclusively on self-reporting of mobile phone ownership, internet access, and internet use and did not attempt in any way to verify these reports (eg, via phone bills, direct observation, or other methods).

### Conclusions

Although health-related internet use statistics are lower than those in the general population, the results showed that the pattern of use is similar. The idea of involving homeless populations in Hungary in the digital health ecosystem is not far-fetched, but a rather viable concept, especially if barriers to access are systematically reduced and the enablers of use strengthened.

During the development of a digital ecosystem, several factors might be considered, such as the role of the institutions providing social and medical services. From an infrastructural point of view, the unavailability and poor affordability of devices and subscriptions and the lack of publicly available free Wi-Fi hotspots were mentioned as barriers to digital technological access. All these factors might be improved by making adequate changes, enabling more Wi-Fi hotspots and installing more publicly available computers in social institutions. In addition, an internet service scheme specifically designed for the homeless population (eg, prepaid services available for medical purposes) could facilitate a shift toward better digital health.

It is important to note that despite all the barriers to accessing digital technologies, our research identified a digitally engaged homeless subgroup, whose members are actively using digital tools for health purposes. With a deeper analysis of this group, characteristics, motivations, and potentials for widening access and use could be delineated, and this group could form a baseline for holistic and appropriate digital public health interventions.

Our preliminary analysis in this group already showed that the characteristics of accommodation also play a role in assessing the accessibility of homeless populations to digital health services. People experiencing homelessness with a more stable housing solution tend to be more open to digital technology and have more access to their own digital resources than others with less stable conditions. This information might be fruitfully used when planning further complex and holistic digital health programs for homeless populations centered on institutions as already available resources for further development.

## Appendix

Multimedia Appendix 1 Digital health access and literacy survey for people experiencing homelessness.

## Abbreviations

HCSOM: Hungarian Charity Service of the Order of Malta
mHealth: mobile health
