# Supplementary material for: Digital Technology Access and Health-Related Internet Use Among People Experiencing Homelessness in Hungary: Quantitative Survey
Source: J Med Internet Res. 2022 Oct 19;24(10):e38729. doi: 10.2196/38729 (PMC9631172; doi:10.2196/38729)
Supplement: Multimedia Appendix 1 [file jmir_v24i10e38729_app1.docx]

1. **ANNEX:**

**DIGITAL HEALTH ACCESS AND LITERACY SURVEY FOR PEOPLE EXPERIENCING HOMELESSNESS**

**DEMOGRAPHICS AND GENERAL INFORMATION**

**Gender: Female / Male Date of birth:**

**1. Do you consider yourself homeless?** NO YES (If yes, since when) YEAR:

**2. What is your highest completed level of education?**

primary school or lower vocational training high school university, college

**3. How frequently do you visit a medical doctor/ do you use medical services?**

Several times a month 1-2 occasions per month Every six months Yearly or less frequently

**4. Do you have any chronic disease or a long-term health problem? By long-term, we mean a problem which has lasted 6 months or longer.**

Yes, and that is No I don’t know

**5. How would you assess the state of your own health?**

Very good Rather good Average Rather poor Very poor I don’t know

**HEALTH LITERACY**

6. Have you ever had any trouble reading health-related documents? Yes/No

7. Did you ask for any help filling in official documents or forms in the last year? Yes/No

**8. How would you assess your general knowledge of health-related topics? Would you**

**say it is...?**

Very good Rather good Average Rather poor Very poor I don’t know

**9. When you need to search for health-related information, what do you usually do?**

You ask your doctor

You ask a pharmacist

You ask your friends/ relatives

You ask someone in the shelter

You search for it online

Other

**10. When trying to access general information on health-related topics or**

**ways to improve your health, which of the following types of information did you look for?**

Information on diseases (symptoms, treatments, interventions)

Results of medical exams

Information on pregnancy, childbirth and early infancy

Information on prescribed pharmaceuticals, medical treatments

Testimonials or experiences from other patients

Other

**MOBILE PHONE**

11. Do you have a mobile phone? Yes / No

12. If yes, is your mobile phone a smartphone (e.g. able to run apps? Yes / No

13. If yes, do you share your phone with others (e.g. family member, friend)? Yes / No, I’m the only user

**INTERNET USE**

14. Have you ever used the Internet for any purpose? Yes/ No

15. If yes, have you used it in the last six months? Yes / No

**16. If you have used the Internet, approximately how often have you used it?**

Every day Several days a week Every week Every second week Once a month Less than once month

**17. How do you currently access the Internet? (circle all the responses which apply)**

Own SMART phone using a data contract

Own SMART phone using a pay as you go facility

Own SMART phone using free wifi access

Computer at hostel or day centre

Own computer

Other

**18. To what extent do you consider yourself a competent Internet user?**

Very competent Rather competent Average Less competent Not at all competent

**19. What do you use the Internet for? (circle all the responses which apply)**

Official business e.g. benefits, job applications

Personal email

Social media (e.g. Facebook, Twitter, Instagram etc)

Playing Games

Viewing things on Youtube

Finding local health / mental health / addictions / homelessness services

Other

**20. Have you ever used the Internet for health reasons? Yes / No**

**21. If yes, what form does this take? (circle all the responses which apply)**

Look up health advice

Look up medication advice

Finding health services for homeless persons

Talking to others about health-related topics

Other

**22. What barriers, if any, restrict your Internet use? (circle all the responses which apply)**

Nothing restricts my internet use

I have Internet access but I have no skills to use the Internet

I have a smartphone but I have no data contract/ money to pay as you go

There are not enough places to access free WIFI

I have no smartphone

There is not enough publicly available computer (e.g. at shelters)

There is no place I can access the Internet

**23. What would help you use the Internet more? (circle all the responses which apply)**

Being given an appropriate device – if I had a smartphone, I know I would use it for accessing the internet

Better access – I have a smartphone, but there needs to be more free wifi that I

can access and/or I would need a data contract/money to pay as you go

Better access – I currently use computers available at shelters, but there is not enough devices available

More knowledge - I don’t have the necessary skills to use the Internet, it would help if I could get assistance

Not relevant - I currently use the Internet as much as I want to

**24. Do you agree with the following statements?**

You know how to navigate the Internet to find the answers to your health questions

Totally agree Tend to agree Tend to disagree Totally disagree (I don’t know)

It doesn’t cause any trouble to understand the terminology used on the Internet for health-related topics

Totally agree Tend to agree Tend to disagree Totally disagree (I don’t know)

You can distinguish high- quality from low-quality health-related information on the Internet

Totally agree Tend to agree Tend to disagree Totally disagree (I don’t know)

After looking online to find information on health-related topics, you are generally more

confused than before

Totally agree Tend to agree Tend to disagree Totally disagree (I don’t know)

Your research on the Internet helps you improve your knowledge of health- related topics

Totally agree Tend to agree Tend to disagree Totally disagree (I don’t know)

**MOBILE APPLICATIONS**

25. Have you ever used any health-related mobile application? Yes / No

If yes, which one?
